# Supplementary material for: Proximity labeling of axonemal protein CFAP91 identifies EFCAB5 that regulates sperm motility
Source: Nat Commun. 2025 Sep 10;16:8238. doi: 10.1038/s41467-025-63705-7 (PMC12423330; doi:10.1038/s41467-025-63705-7)
Supplement: Supplementary file 1 — Supplementary Information [file 41467_2025_63705_MOESM1_ESM.pdf]

Proximity Labeling of Axonemal Protein CFAP91 Identifies EFCAB5 that Regulates Sperm Motility

Supplementary Information

Supplementary Fig. 1

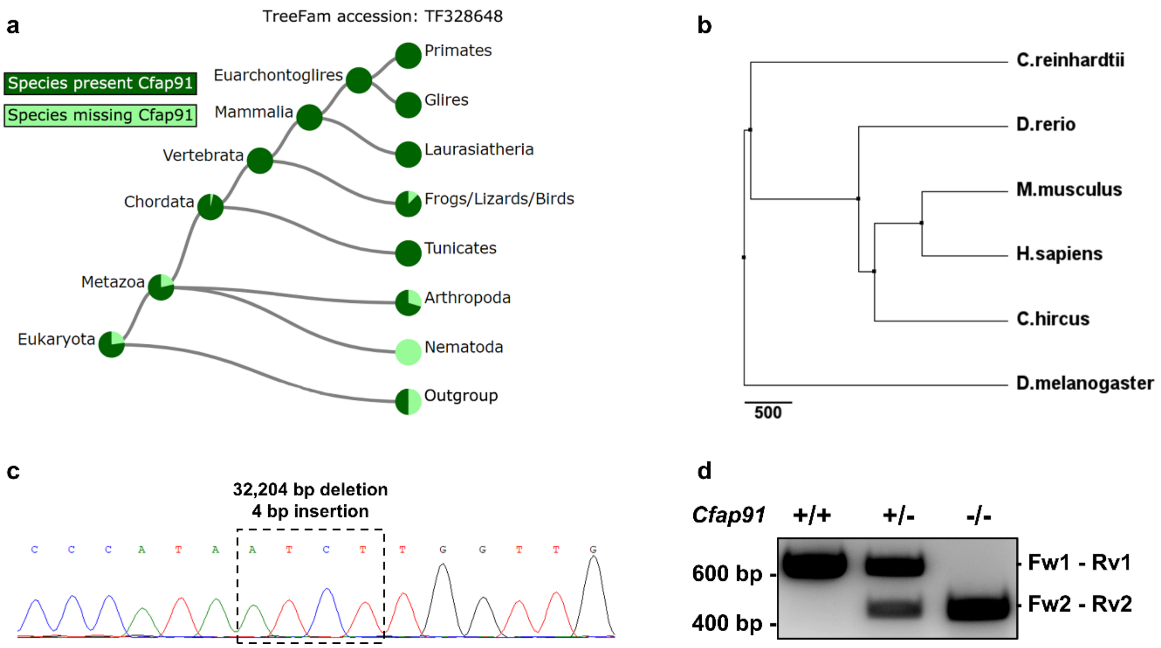

Supplementary Fig. 1: Conservation and ablation of mouse *Cfap91*

a Conservation of *Cfap91* among various species included in the TreeFam database. **b** Phylogenetic analysis using amino acid sequences of CFAP91. **c** A large deletion in the genomic region of *Cfap91* was confirmed by Sanger sequencing. **d** Genomic PCR of *Cfap91* mutant mice. The primers shown in Fig. 1e were used.

Supplementary Fig. 2

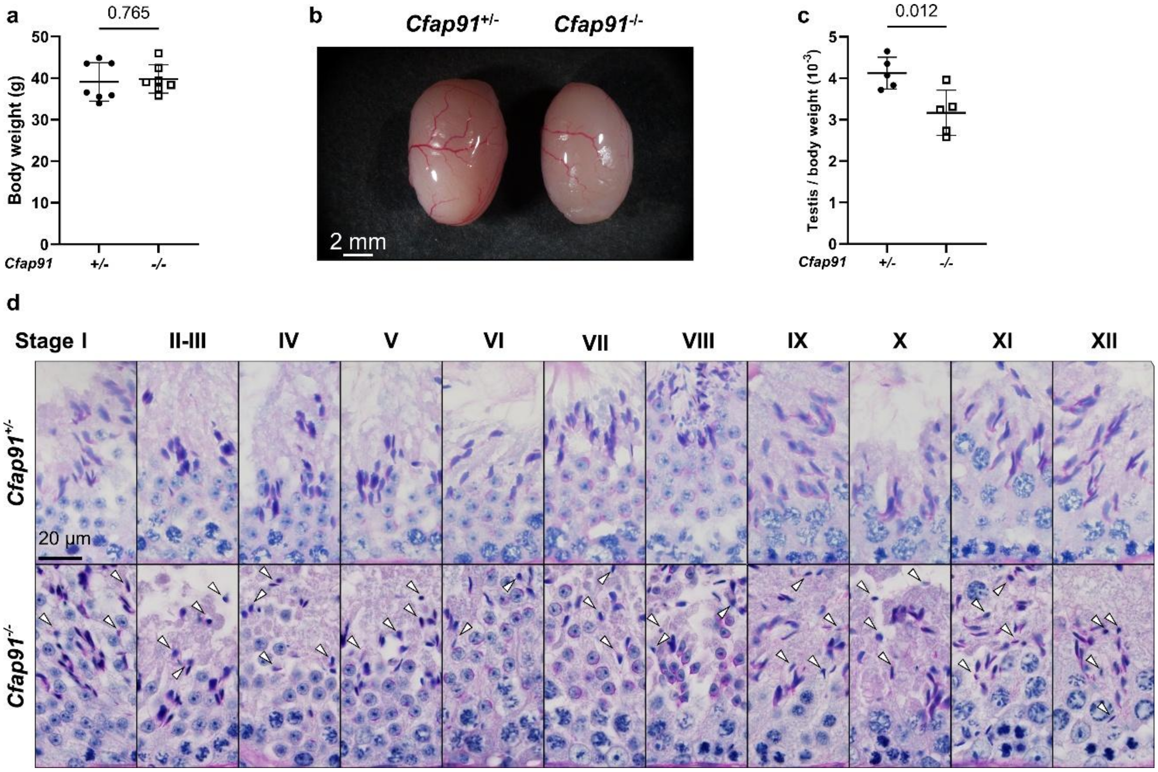

Supplementary Fig. 2: Analyses of *Cfap91*$^{-/-}$ testes

a Body weight of *Cfap91*$^{-/-}$ males was not altered compared to *Cfap91*$^{+/-}$ males (n=7 males for each genotype). Data were presented as mean ± SD. An unpaired two-tailed t-test was performed for statistical analysis. **b** Gross observation of testis from *Cfap91*$^{+/-}$ and *Cfap91*$^{-/-}$ males. **c** Comparison of testis weight standardized to body weight between *Cfap91*$^{+/-}$ and *Cfap91*$^{-/-}$ males (n=5 males for each genotype). Data were presented as mean ± SD. An unpaired two-tailed t-test was performed for statistical analysis. **d** Testis sections of *Cfap91*$^{+/-}$ and *Cfap91*$^{-/-}$ males. Images were categorized according to the stage of the seminiferous tubules. Abnormal spermatids were indicated by arrowheads.

Supplementary Fig. 3

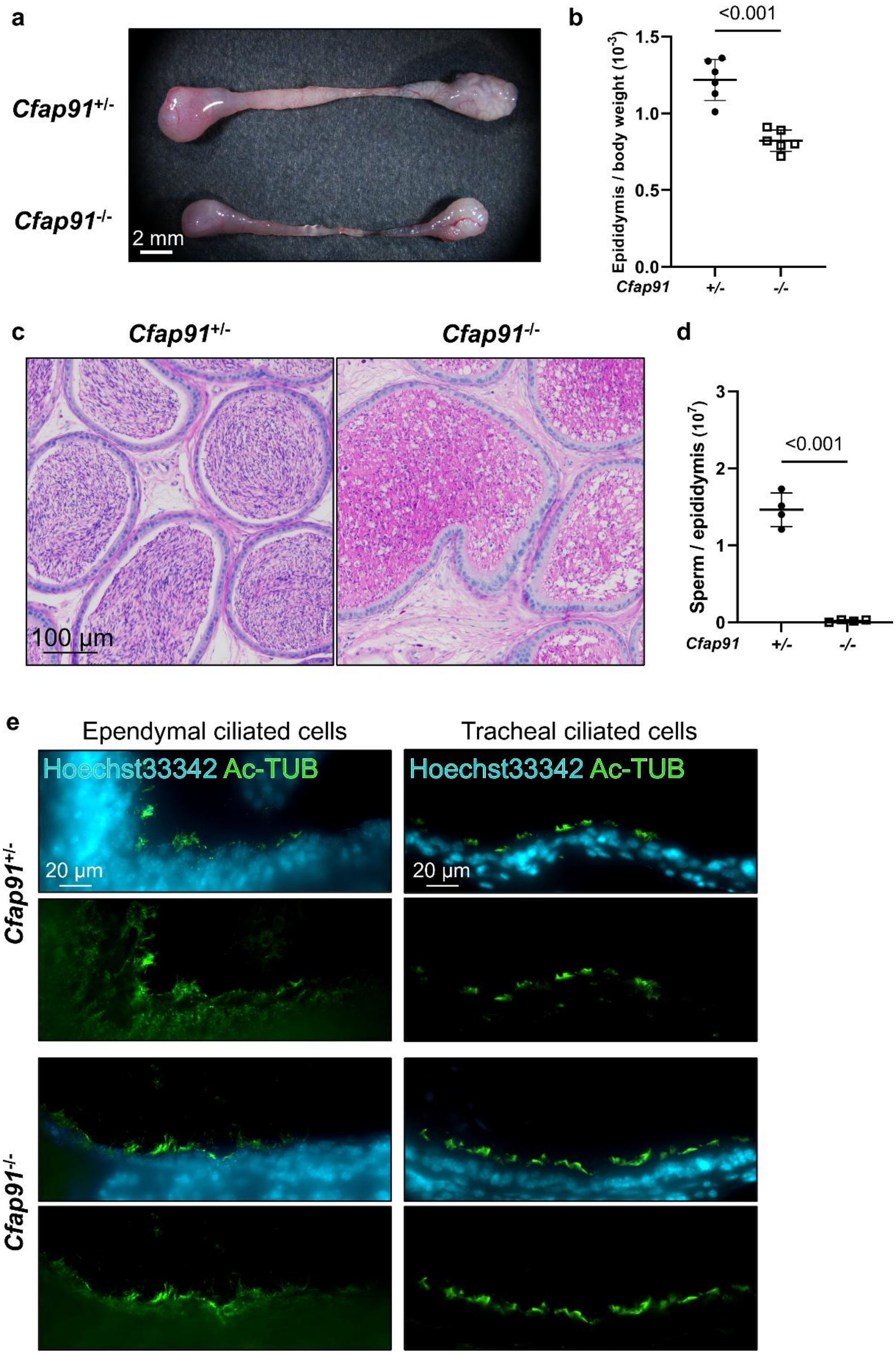

Supplementary Fig. 3: Analyses of *Cfap91$^{-/-}$* epididymis

a Gross observation of epididymis from *Cfap91$^{+/-}$* and *Cfap91$^{-/-}$* males. **b** Comparison of epididymis weight standardized to body weight between *Cfap91$^{+/-}$* and *Cfap91$^{-/-}$* males (n=6 males for each genotype). Data were presented as mean ± SD. An unpaired two-tailed t-test was performed for statistical analysis. *P* = 7.3E-5. **c** Cauda epididymal sections of *Cfap91$^{+/-}$* and *Cfap91$^{-/-}$* males. **d** The numbers of cauda epididymal spermatozoa were compared between *Cfap91$^{+/-}$* and *Cfap91$^{-/-}$* males (n=4 males for each genotype). Data were presented as mean ± SD. An unpaired two-tailed t-test was performed for statistical analysis. *P* = 1.2E-5. **e** Immunohistochemistry on the sections of mouse brain and trachea from *Cfap91$^{+/-}$* and *Cfap91$^{-/-}$* males, with an anti-acetylated tubulin antibody labeling motile cilia (green).

Supplementary Fig. 4

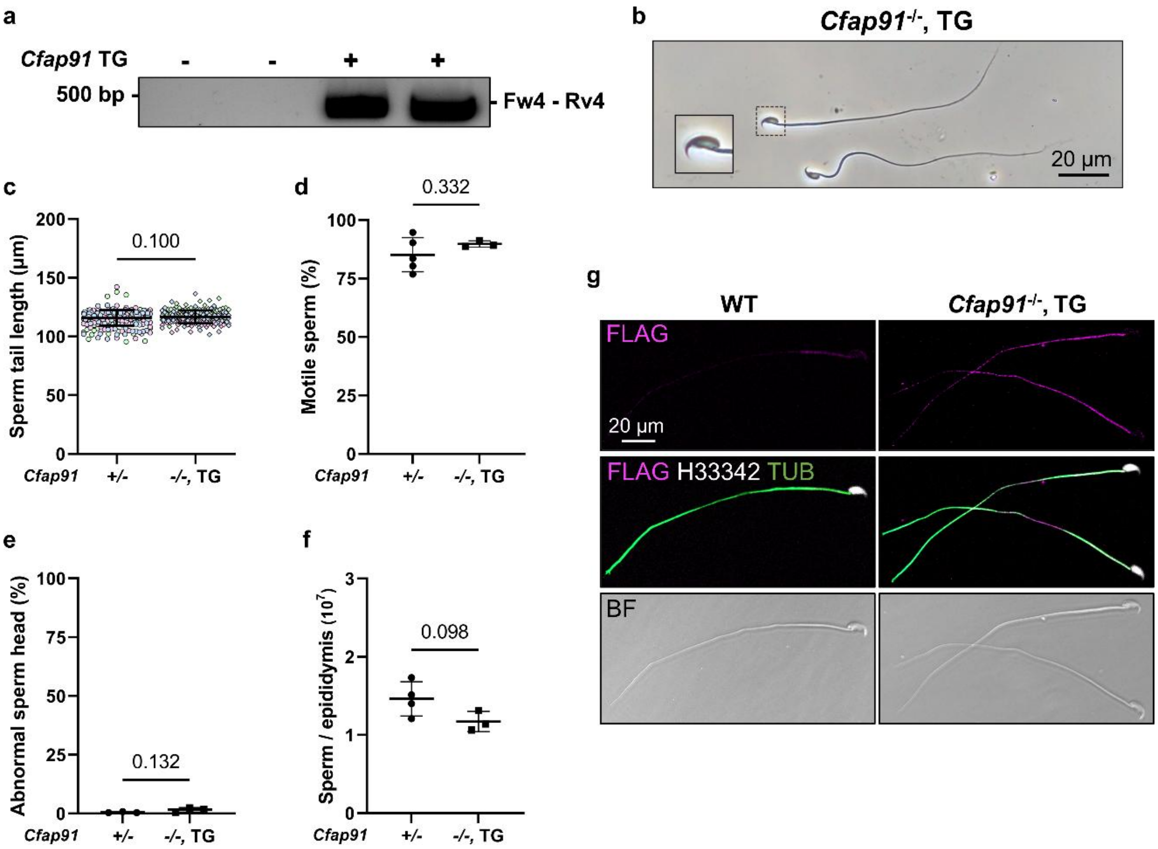

Supplementary Fig. 4: Analyses of *Cfap91* TG males

a Genomic PCR using primers shown in Fig. 4a for *Cfap91* TG mice. **b** Phase contrast images of cauda epididymal spermatozoa from *Cfap91*$^{-/-}$ TG males. **c–f** Comparison of length of the sperm tail (n=3 males for each genotype) (**c**), percentage of motile spermatozoa (n=5 males for *Cfap91*$^{+/-}$ males and n=3 males for *Cfap91*$^{-/-}$ TG males) (**d**), percentage of abnormal sperm head (n=3 males for each genotype) (**e**), and the number of cauda epididymal spermatozoa (n=4 males for *Cfap91*$^{+/-}$ males and n=3 males for *Cfap91*$^{-/-}$ TG males) (**f**). The same data of *Cfap91*$^{+/-}$ males from Fig. 2b-2d, and Supplementary Fig. 3d were used. Data were presented as mean ± SD. An unpaired two-tailed t-test was performed for statistical analysis. **g** Immunocytochemistry of cauda epididymal spermatozoa from *Cfap91*$^{-/-}$ TG males. CFAP91 is localized along the sperm tail.

Supplementary Fig. 5

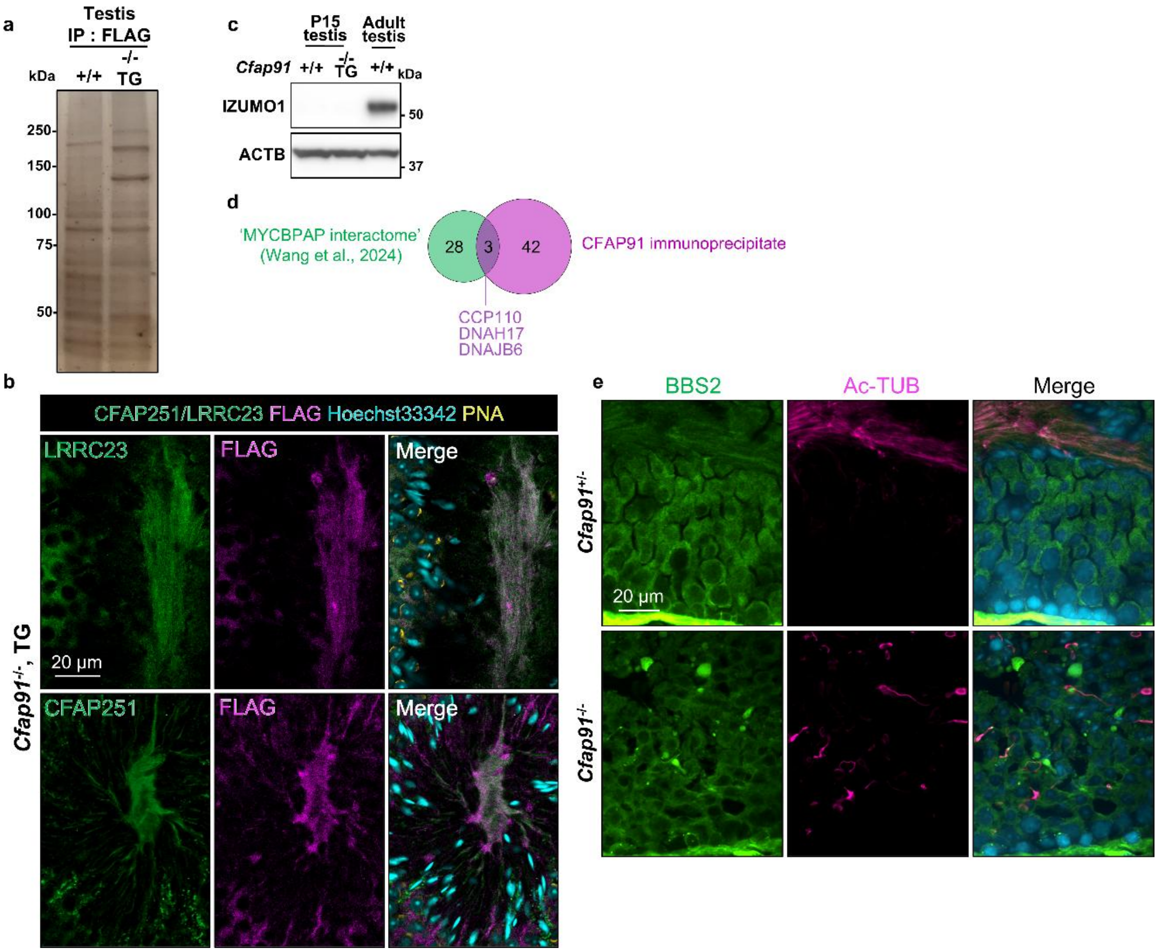

Supplementary Fig. 5: immunoprecipitates of CFAP91

a Silver staining of the IP product. IP was performed using the testicular lysate of *Cfap91*$^{-/-}$ TG males. **b** Immunohistochemistry on testicular sections of *Cfap91*$^{-/-}$ TG males. CFAP91 (FLAG) was colocalized with both CFAP251 and LRRC23. **c** Immunoblotting using testicular lysates. IZUMO1 signal was found in adult testis but not P15 testis. ACTB served as a loading control. **d** Venn diagram showed proteins significantly enriched in both CFAP91 and MYCBPAP immunoprecipitates. **e** Immunohistochemistry on the testicular sections from *Cfap91*$^{+/-}$ and *Cfap91*$^{-/-}$ males with an anti-BBS2 antibody.

Supplementary Fig. 6

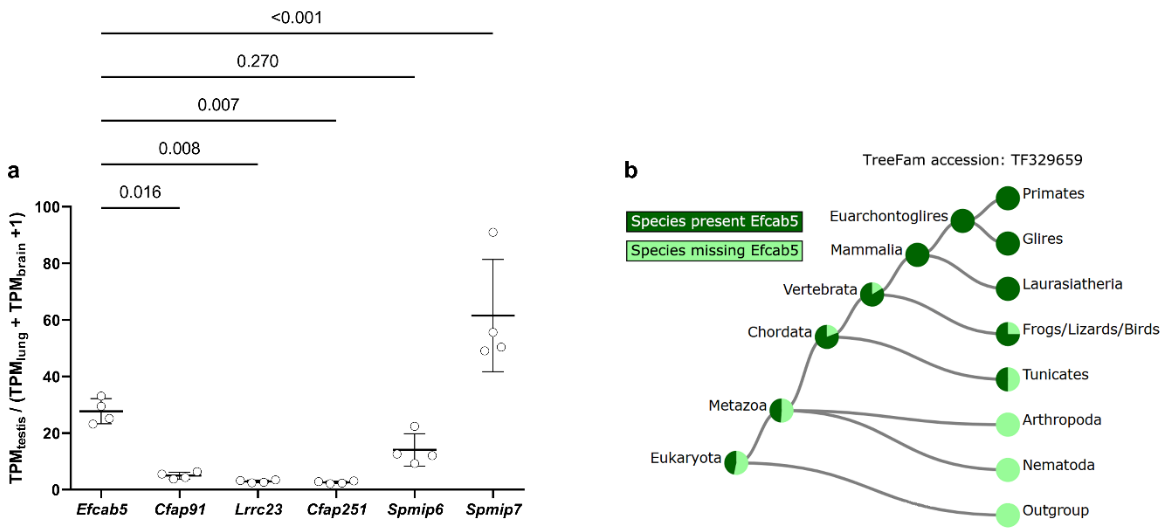

Supplementary Fig. 6: Expression in mice and conservation of EFCAB5

a Comparison of the testicular expression ratio of genes. SPMIP6 and SPMIP7 are two proteins only found in the bovine sperm axoneme but not in the tracheal ciliary axoneme. The testicular expression ratio was calculated as [TPM_{testis} / (TPM_{lung} + TPM_{brain} +1)]. One-way ANOVA was used for statistical analysis (n=4 studies for each gene). *P* = 3.7E-4 when *Efcab5* and *Spmip7* were compared. **b** Conservation of EFCAB5 among various species included in the TreeFam database.

Supplementary Fig. 7

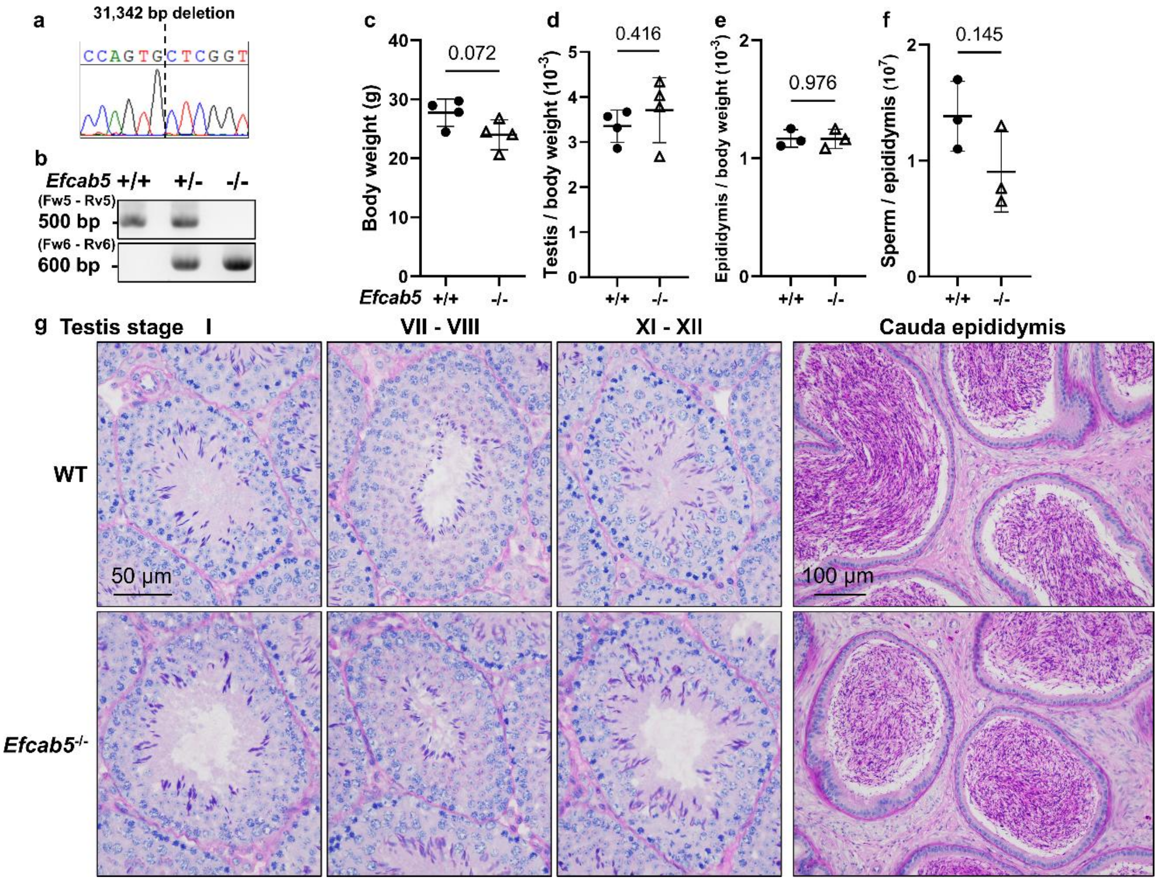

Supplementary Fig. 7: Analyses of *Efcab5*$^{-/-}$ mice

a Large deletion of the genomic region of *Efcab5* was confirmed by Sanger sequencing. **b** Genomic PCR of *Efcab5* mutant mice. The primers shown in Fig. 7a were used. **c–f** Comparison of body weight (n=4 males for each genotype) (**c**), testis weight (n=4 males for each genotype) (**d**), epididymis weight (n=3 males for each genotype) (**e**), and the number of cauda epididymal spermatozoa (n=3 males for each genotype) (**f**) between *Efcab5*$^{+/+}$ and *Efcab5*$^{-/-}$ males. Data were presented as mean ± SD. An unpaired two-tailed t-test was performed for statistical analysis. **g** Testis and cauda epididymis sections from WT and *Efcab5*$^{-/-}$ males. Images of the testis were categorized according to the stage of the seminiferous tubules.

Supplementary Fig. 8

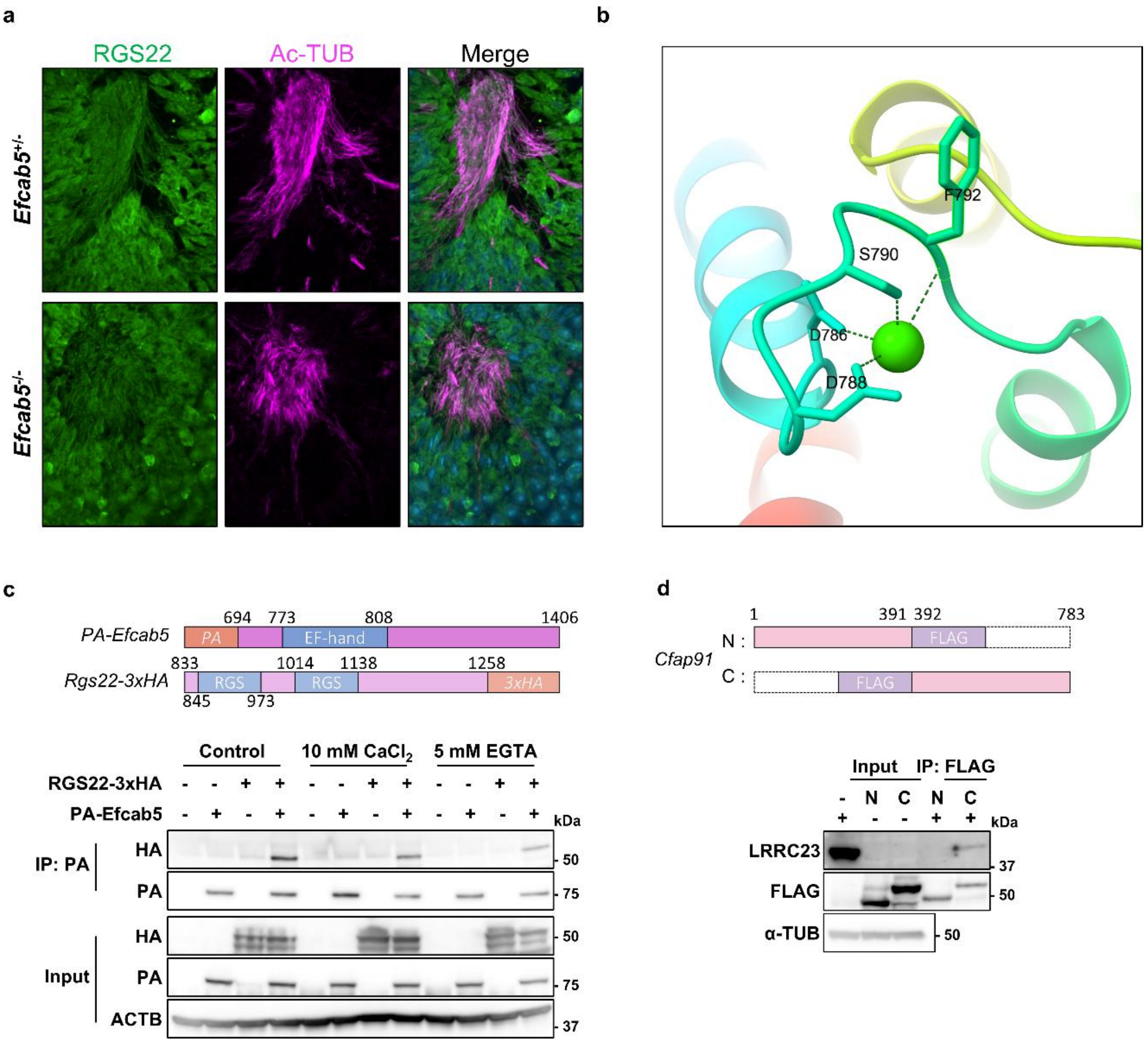

Supplementary Fig. 8: Analyses of RGS22, EFCAB5, and CFAP91

a Immunohistochemistry on testicular sections from *Efcab5*$^{+/-}$ and *Efcab5*$^{-/-}$ males with an anti-RGS22 antibody. **b** The structure of the complex formed by EFCAB5 with Ca^{2+} was predicted by AlphaFold. Predicted template modeling (pTM) = 0.75, interface pTM (ipTM) = 0.84. **c** The fractions of EFCAB5 and RGS22 that were predicted to show interaction in the previous study[35] were cloned. EFCAB5 and RGS22 were heterologously expressed in HEK293T cells, and EGTA or $CaCl_2$ was added to the lysates. IP was performed using an anti-PA antibody, and immunoblotting was performed using the IP product. RGS indicates the regulation of G-protein signaling (RGS) domain, while EF-hand indicates the EF-hand domain that possesses calcium-

binding ability. **d** The N-terminus and C-terminus of CFAP91 were expressed in HEK293T cells and collected by IP. IP products were mixed with *Cfap91*$^{-/-}$ testicular lysate to pull down associated proteins.
