# Supplementary material for: Proximity labeling of axonemal protein CFAP91 identifies EFCAB5 that regulates sperm motility
Source: Nat Commun. 2025 Sep 10;16:8238. doi: 10.1038/s41467-025-63705-7 (PMC12423330; doi:10.1038/s41467-025-63705-7)
Supplement: Supplementary file 3 — Description of Additional Supplementary Files [file 41467_2025_63705_MOESM3_ESM.pdf]

Description of Additional Supplementary Files

File Name: Supplementary Data 1
Description: **Proteins identified in the IP product using adult testicular lysates.**
An unpaired two-tailed t-test was performed for statistical analysis.

File Name: Supplementary Data 2
Description: **Categorization of CFAP91 immunoprecipitates.**

File Name: Supplementary Data 3
Description: **Proteins identified in the pull down product using Cfap91 KO TG spermatozoa.**

File Name: Supplementary Data 4
Description: **Sequence of primers and guide RNAs used in this study.**

File Name: Supplementary Data 5
Description: **Antibodies used in this study.**

File Name: Supplementary Movie 1
Description: Spermatozoa were videotaped at 200 frames per second and the movie is played at 20 frames per second.
