# Supplementary material for: Proximity labeling of axonemal protein CFAP91 identifies EFCAB5 that regulates sperm motility
Source: Nat Commun. 2025 Sep 10;16:8238. doi: 10.1038/s41467-025-63705-7 (PMC12423330; doi:10.1038/s41467-025-63705-7)
Supplement: Supplementary file 10 — Reporting Summary [file 41467_2025_63705_MOESM10_ESM.pdf]

nature portfolio

Corresponding author(s): Haruhiko Miyata, Masahito Ikawa

Last updated by author(s): August 12, 2025

Reporting Summary

Nature Portfolio wishes to improve the reproducibility of the work that we publish. This form provides structure for consistency and transparency in reporting. For further information on Nature Portfolio policies, see our Editorial Policies and the Editorial Policy Checklist.

Please do not complete any field with "not applicable" or n/a. Refer to the help text for what text to use if an item is not relevant to your study.
For final submission: please carefully check your responses for accuracy; you will not be able to make changes later.

Statistics

For all statistical analyses, confirm that the following items are present in the figure legend, table legend, main text, or Methods section.

n/a	Confirmed	
☐	☒	The exact sample size (*n*) for each experimental group/condition, given as a discrete number and unit of measurement
☐	☒	A statement on whether measurements were taken from distinct samples or whether the same sample was measured repeatedly
☐	☒	The statistical test(s) used AND whether they are one- or two-sided<br>*Only common tests should be described solely by name; describe more complex techniques in the Methods section.*
☒	☐	A description of all covariates tested
☒	☐	A description of any assumptions or corrections, such as tests of normality and adjustment for multiple comparisons
☐	☒	A full description of the statistical parameters including central tendency (e.g. means) or other basic estimates (e.g. regression coefficient) AND variation (e.g. standard deviation) or associated estimates of uncertainty (e.g. confidence intervals)
☐	☒	For null hypothesis testing, the test statistic (e.g. *F*, *t*, *r*) with confidence intervals, effect sizes, degrees of freedom and *P* value noted<br>*Give P values as exact values whenever suitable.*
☒	☐	For Bayesian analysis, information on the choice of priors and Markov chain Monte Carlo settings
☒	☐	For hierarchical and complex designs, identification of the appropriate level for tests and full reporting of outcomes
☒	☐	Estimates of effect sizes (e.g. Cohen's *d*, Pearson's *r*), indicating how they were calculated

Our web collection on statistics for biologists contains articles on many of the points above.

Software and code

Policy information about availability of computer code

Data collection	Sperm motility data was collected using the CEROS II (software version 1.5; Hamilton Thorne Biosciences, Beverly, MA, USA) sperm analysis system.
Data analysis	Proteomic data was analyzed by Scaffold 5 (Proteome Software, Portland, OR). Statistical analyses were performed using GraphPad Prism 9 (GraphPad Software, MA, USA).

For manuscripts utilizing custom algorithms or software that are central to the research but not yet described in published literature, software must be made available to editors and reviewers. We strongly encourage code deposition in a community repository (e.g. GitHub). See the Nature Portfolio guidelines for submitting code & software for further information.

Data

Policy information about availability of data

All manuscripts must include a data availability statement. This statement should provide the following information, where applicable:

- Accession codes, unique identifiers, or web links for publicly available datasets
- A description of any restrictions on data availability
- For clinical datasets or third party data, please ensure that the statement adheres to our policy

The authors declare that the data that support the findings of this study are available from the corresponding author upon request. Proteomic data is available with accession codes JPST003879 [https://repository.jpostdb.org/entry/JPST003879.2] and PXD065243 [https://proteomecentral.proteomexchange.org/cgi/GetDataset?ID=PXD065243]. Cryo-EM density maps and fitted model were retrieved from previous studies with accession codes EMDB-35888 [https://www.ebi.ac.uk/emdb/EMD-35888], EMDB-50664 [https://www.ebi.ac.uk/pdbe/entry/emdb/EMD-50664] and 9FQR [https://doi.org/10.2210/pdb9fqr/pdb].

Research involving human participants, their data, or biological material

Policy information about studies with human participants or human data. See also policy information about sex, gender (identity/presentation), and sexual orientation and race, ethnicity and racism.

Reporting on sex and gender	Males
Reporting on race, ethnicity, or other socially relevant groupings	Asian
Population characteristics	25 - 45 years old healthy individuals
Recruitment	Volunteers from The University of Osaka
Ethics oversight	The experiments using human samples were approved by the Ethics Committee of the Research Institute for Microbial Diseases, Osaka University (#28-4-2).

Note that full information on the approval of the study protocol must also be provided in the manuscript.

Field-specific reporting

Please select the one below that is the best fit for your research. If you are not sure, read the appropriate sections before making your selection.

☒ Life sciences ☐ Behavioural & social sciences ☐ Ecological, evolutionary & environmental sciences

For a reference copy of the document with all sections, see nature.com/documents/nr-reporting-summary-flat.pdf

Life sciences study design

All studies must disclose on these points even when the disclosure is negative.

Sample size	Sample sizes were determined based on published studies in the field or our previous experiences. No statistics was used to predetermine the sample size.
Data exclusions	No samples or animals were excluded. Also, the criteria were not pre-established in experiments.
Replication	All experiments were performed using at least three biological replicas.
Randomization	Samples are defined by their unique genotypes. Therefore, no sample randomization was performed.
Blinding	The investigators were not blinded for group allocation.

Behavioural & social sciences study design

All studies must disclose on these points even when the disclosure is negative.

Study description	
Research sample	
Sampling strategy	
Data collection	
Timing	
Data exclusions	
Non-participation	
Randomization	

Ecological, evolutionary & environmental sciences study design

All studies must disclose on these points even when the disclosure is negative.

Study description	
Research sample	
Sampling strategy	
Data collection	
Timing and spatial scale	
Data exclusions	
Reproducibility	
Randomization	
Blinding	

Did the study involve field work? ☐ Yes ☐ No

Field work, collection and transport

Field conditions	
Location	
Access & import/export	
Disturbance	

Reporting for specific materials, systems and methods

We require information from authors about some types of materials, experimental systems and methods used in many studies. Here, indicate whether each material, system or method listed is relevant to your study. If you are not sure if a list item applies to your research, read the appropriate section before selecting a response.

Materials & experimental systems

n/a	Involved in the study	
☐	☒	Antibodies
☐	☒	Eukaryotic cell lines
☒	☐	Palaeontology and archaeology
☐	☒	Animals and other organisms
☒	☐	Clinical data
☒	☐	Dual use research of concern
☒	☐	Plants

Methods

n/a	Involved in the study	
☒	☐	ChIP-seq
☒	☐	Flow cytometry
☒	☐	MRI-based neuroimaging

Antibodies

Antibodies used	Anti-1D4 (From Dr. Martin M. Matzuk), anti-Acetylated tubulin (Sigma-Aldrich , T7451), anti-Acetylated tubulin (Cell Signaling Technology , D20G3), anti-ACTB (Abcam, ab6276), anti-AKAP3 (Proteintech, 13907-1-AP), anti-AKAP4 (BD Biosciences , 611564), anti-Alpha-tubulin (Sigma-Aldrich , T6074), anti-BASIGIN (This lab), anti-BBS2 (Santa Cruz, sc-365355), anti-CFAP251 (Atlas Antibodies , HPA040005), anti-EFCAB5 (This study), anti-EFCAB5 (human) (Proteintech, 24296-1-AP), anti-EGFP (This lab), anti-FLAG (MBL, PM020), anti-FLAG (Sigma-Aldrich , F1804), anti-HA (MBL, M180-3), anti-IFT140 (Proteintech, 17460-1-AP), anti-IZUMO1 (This lab, 64-125), anti-LRRC23 (This lab), anti-PA (FUJIFILM Wako Pure Chemical Corp., NZ-1), anti-RGS22 (Invitrogen, PA5-104095), Streptavidin-HRP (Proteintech, SA00001-0), HRP-cojugated anti-mouse IgG (Jackson ImmunoResearch Inc., 115-036-082), HRP-cojugated anti-rabbit IgG (Jackson ImmunoResearch Inc., 111-036-045), HRP-cojugated anti-rat IgG (Jackson ImmunoResearch Inc., 112-035-167), Alexa Fluor 488 conjugated anti-mouse IgG (Thermo Fisher Scientific, A11017), Alexa Fluor 546 conjugated anti-mouse IgG (Thermo Fisher Scientific, A11018), Alexa Fluor 647 conjugated anti-mouse IgG (Thermo Fisher Scientific, A21235), Alexa Fluor 488 conjugated anti-rabbit IgG (Thermo Fisher Scientific, A11070), Alexa Fluor 546 conjugated anti-rabbit IgG (Thermo Fisher Scientific, A11071), Alexa Fluor 647 conjugated anti-rabbit IgG (Thermo Fisher Scientific, A21245), Alexa Fluor 488 conjugated anti-rat IgG (Thermo Fisher Scientific, A11006), Alexa Fluor 546 conjugated anti-rat IgG (Thermo Fisher Scientific, A11081), Alexa Fluor 647 conjugated anti-rat IgG (Thermo Fisher Scientific, A21247) were used in this study.
Validation	All antibodies except anti-EFCAB5 antibody were validated by previous papers and manufacturers. Anti-EFCAB5 antibody was validated by immunoblotting in Fig. 7d in this study.

Eukaryotic cell lines

Policy information about cell lines and Sex and Gender in Research

Cell line source(s)	HEK293T cell line was gifted from Dr. Verma Lab (The Salk Institute).
Authentication	Cell lines were authenticated based on their morphology and growth.
Mycoplasma contamination	We confirmed all the cells used in this study were negative for mycoplasma contamination.
Commonly misidentified lines (See ICLAC register)	No commonly misidentified cell lines were used in this study.

Palaeontology and Archaeology

Specimen provenance	
Specimen deposition	
Dating methods	

☐ Tick this box to confirm that the raw and calibrated dates are available in the paper or in Supplementary Information.

Ethics oversight	

Note that full information on the approval of the study protocol must also be provided in the manuscript.

Animals and other research organisms

Policy information about studies involving animals; ARRIVE guidelines recommended for reporting animal research, and Sex and Gender in Research

Laboratory animals	BDF1 and ICR mice were purchased from Japan SLC (Shizuoka, Japan) and CLEA Japan (Tokyo, Japan). Mice in the 129X1/SvJmsSlc background were purchased from Japan SLC. Mice were acclimated to 12-h-light/12-h-dark cycle.
Wild animals Reporting	This study did not involve wild animals.
on sex Field-collected	Sex of animals used are indicated in each experiment.
samples Ethics	This study did not involve samples collected from the field.
oversight	All mouse experiments were approved by the Animal Care and Use Committee at the Research Institute for Microbial Diseases, Osaka University (Approval numbers: #Biken-AP-H30-01 and #Biken-AP-R03-01).

Note that full information on the approval of the study protocol must also be provided in the manuscript.

Clinical data

Policy information about clinical studies

All manuscripts should comply with the ICMJE guidelines for publication of clinical research and a completed CONSORT checklist must be included with all submissions.

Clinical trial registration	
Study protocol	
Data collection	
Outcomes	

Dual use research of concern

Policy information about dual use research of concern

Hazards

Could the accidental, deliberate or reckless misuse of agents or technologies generated in the work, or the application of information presented in the manuscript, pose a threat to:

No	Yes	
☐	☐	Public health
☐	☐	National security
☐	☐	Crops and/or livestock
☐	☐	Ecosystems
☐	☐	Any other significant area

Experiments of concern

Does the work involve any of these experiments of concern:

No	Yes	
☐	☐	Demonstrate how to render a vaccine ineffective
☐	☐	Confer resistance to therapeutically useful antibiotics or antiviral agents
☐	☐	Enhance the virulence of a pathogen or render a nonpathogen virulent
☐	☐	Increase transmissibility of a pathogen
☐	☐	Alter the host range of a pathogen
☐	☐	Enable evasion of diagnostic/detection modalities
☐	☐	Enable the weaponization of a biological agent or toxin
☐	☐	Any other potentially harmful combination of experiments and agents

Plants

Seed stocks

Novel plant genotypes

Authentication

ChIP-seq

Data deposition

☐ Confirm that both raw and final processed data have been deposited in a public database such as GEO.

☐ Confirm that you have deposited or provided access to graph files (e.g. BED files) for the called peaks.

Data access links
May remain private before publication.

Files in database submission

Genome browser session
(e.g. UCSC)

Methodology

Replicates

Sequencing depth

Antibodies

Peak calling parameters

Data quality

Software

Flow Cytometry

Plots

Confirm that:

☐ The axis labels state the marker and fluorochrome used (e.g. CD4-FITC).

☐ The axis scales are clearly visible. Include numbers along axes only for bottom left plot of group (a 'group' is an analysis of identical markers).

☐ All plots are contour plots with outliers or pseudocolor plots.

☐ A numerical value for number of cells or percentage (with statistics) is provided.

Methodology

Sample preparation

Instrument

Software

Cell population abundance

Gating strategy

☐ Tick this box to confirm that a figure exemplifying the gating strategy is provided in the Supplementary Information.

Magnetic resonance imaging

Experimental design

Design type

Design specifications

Behavioral performance measures

Imaging type(s)

Field strength

Sequence & imaging parameters

Area of acquisition

Diffusion MRI ☐ Used ☐ Not used

Preprocessing

Preprocessing software

Normalization

Normalization template

Noise and artifact removal

Volume censoring

Statistical modeling & inference

Model type and settings

Effect(s) tested

Specify type of analysis: ☐ Whole brain ☐ ROI-based ☐ Both

Statistic type for inference

(See Eklund et al. 2016)

Correction

Models & analysis

n/a	Involved in the study
☐	☐ Functional and/or effective connectivity
☐	☐ Graph analysis
☐	☐ Multivariate modeling or predictive analysis

Functional and/or effective connectivity

Graph analysis

Multivariate modeling and predictive analysis

This checklist template is licensed under a Creative Commons Attribution 4.0 International License, which permits use, sharing, adaptation, distribution and reproduction in any medium or format, as long as you give appropriate credit to the original author(s) and the source, provide a link to the Creative Commons license, and indicate if changes were made. The images or other third party material in this article are included in the article's Creative Commons license, unless indicated otherwise in a credit line to the material. If material is not included in the article's Creative Commons license and your intended use is not permitted by statutory regulation or exceeds the permitted use, you will need to obtain permission directly from the copyright holder. To view a copy of this license, visit http://creativecommons.org/licenses/by/4.0/
